# Crystal structure of di­bromido­tetra­kis(propan-2-ol-κ*O*)nickel(II)

**DOI:** 10.1107/S2056989015023555

**Published:** 2015-12-19

**Authors:** Yaokang Lv, Mingxian Liu, Lvlv Ji, Cheng Zhang, Mi Ouyang

**Affiliations:** a Department of Chemistry, Tongji University, Shanghai 200092, People’s Republic of China; b College of Chemical Engineering, Zhejiang University of Technology, 310014 Hangzhou, People’s Republic of China

**Keywords:** crystal structure, nickel(II) complex, iso­propanol ligand

## Abstract

The asymmetric unit of the mononuclear title complex, [NiBr_2_(C_3_H_8_O)_4_], comprises a Ni^II^ cation located on a centre of inversion, one Br^−^ anion and two propan-2-ol ligands. The Ni^II^ cation exhibits a distorted *trans*-Br_2_O_4_ environment. There are O—H⋯Br hydrogen bonds connecting neighbouring mol­ecules into rows along [100]. These rows are arranged in a distorted hexa­gonal packing and are held together by van der Waals forces only.

## Related literature   

Nickel complexes have attracted attention due to their coordination chemistry and electrochemical properties. For background to such nickel complexes, see: Kapoor *et al.* (2012 ▸); Kant *et al.* (2015 ▸). For similar crystal structures with propan-2-ol ligands coordinating Ni^2+^ cations, see: Veith *et al.* (2008 ▸).
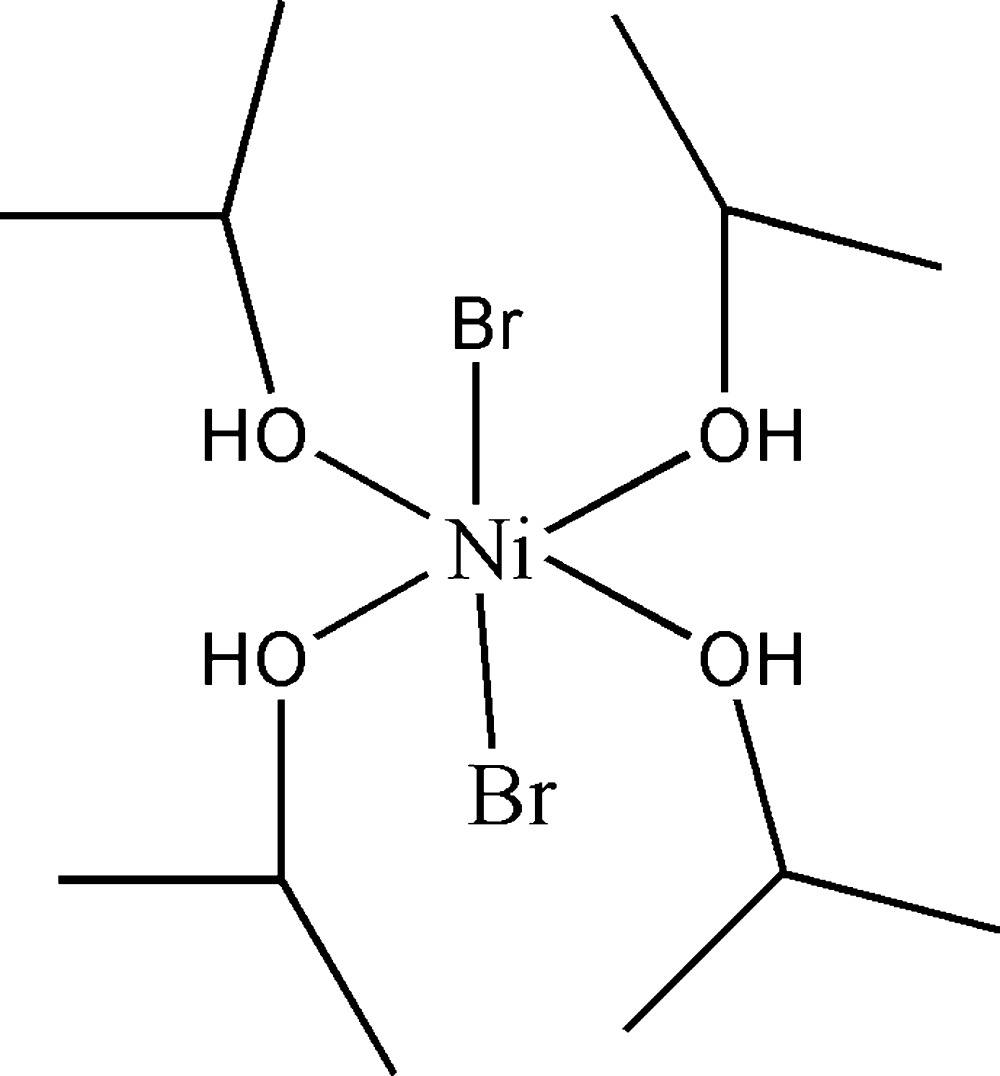


## Experimental   

### Crystal data  

[NiBr_2_(C_3_H_8_O)_4_]
*M* *_r_* = 458.91
Monoclinic, 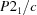

*a* = 5.8341 (7) Å
*b* = 10.4902 (15) Å
*c* = 16.613 (2) Å
β = 97.074 (4)°
*V* = 1009.0 (2) Å^3^
*Z* = 2
Mo *K*α radiation
μ = 4.93 mm^−1^
*T* = 199 K
0.42 × 0.21 × 0.07 mm

### Data collection  

Bruker APEXII CCD diffractometer
Absorption correction: multi-scan (*SADABS*; Bruker, 2006 ▸) *T*_min_ = 0.305, *T*_max_ = 0.710
9106 measured reflections
1770 independent reflections
1451 reflections with *I* > 2σ(*I*)
*R*_int_ = 0.056

### Refinement  

*R*[*F* ^2^ > 2σ(*F* ^2^)] = 0.029
*wR*(*F* ^2^) = 0.071
*S* = 1.01
1770 reflections
94 parameters
2 restraints
H atoms treated by a mixture of independent and constrained refinement
Δρ_max_ = 0.60 e Å^−3^
Δρ_min_ = −0.34 e Å^−3^

### 

Data collection: *APEX2* (Bruker, 2006 ▸); cell refinement: *SAINT* (Bruker, 2006 ▸); data reduction: *SAINT*; program(s) used to solve structure: *SHELXS97* (Sheldrick, 2008 ▸); program(s) used to refine structure: *SHELXL97* (Sheldrick, 2008 ▸); molecular graphics: *DIAMOND* (Brandenburg, 2006 ▸); software used to prepare material for publication: *publCIF* (Westrip, 2010 ▸).

## Supplementary Material

Crystal structure: contains datablock(s) I. DOI: 10.1107/S2056989015023555/wm5238sup1.cif

**Fig. 1.**
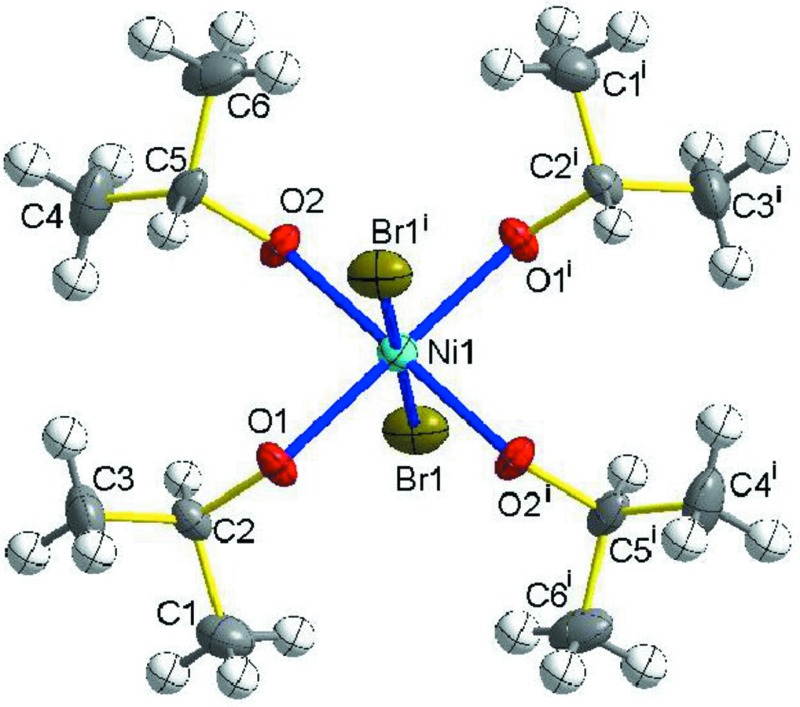
The molecular structure of the title complex. Displacement ellipsoids are drawn at the 30% probability level; H atoms are given as spheres of arbitrary radius. [Symmetry code: (i) 2 - x, -y, -z.]

Structure factors: contains datablock(s) I. DOI: 10.1107/S2056989015023555/wm5238Isup2.hkl

x y z . DOI: 10.1107/S2056989015023555/wm5238fig1.tif
The mol­ecular structure of the title complex. Displacement ellipsoids are drawn at the 30% probability level; H atoms are given as spheres of arbitrary radius. [Symmetry code: (i) 2 − *x*, −*y*, −*z*.]

**Fig. 2.**
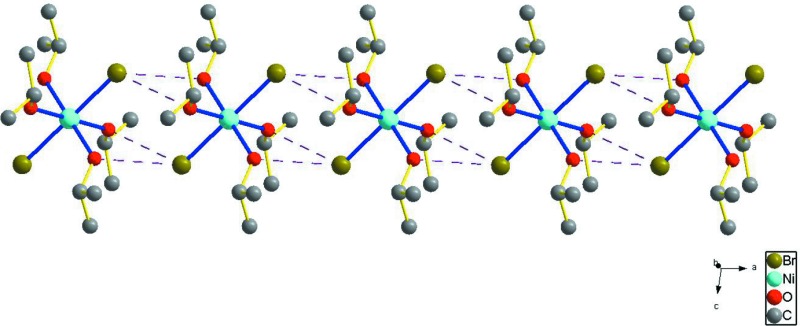
The chain structure of the title complex generated by O—H···Br hydrogen bonds (dotted lines).

. DOI: 10.1107/S2056989015023555/wm5238fig2.tif
The chain structure of the title complex generated by O—H⋯Br hydrogen bonds (dotted lines).

CCDC reference: 1441097

Additional supporting information:  crystallographic information; 3D view; checkCIF report

## Figures and Tables

**Table 1:** Hydrogen-bond geometry (Å, °)

*D*—H⋯*A*	*D*—H	H⋯*A*	*D*⋯*A*	*D*—H⋯*A*
O1—H1⋯Br1^i^	0.81 (2)	2.58 (2)	3.372 (2)	166 (4)
O2—H2⋯Br1^ii^	0.83 (2)	2.51 (2)	3.315 (2)	165 (3)

Symmetry codes: (i) 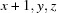; (ii) 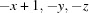.

## supplementary crystallographic information

### S1. Synthesis and crystallization

Anhydrous NiBr_2_ and iso­propanol were purchased from Sigma-Aldrich. The title complex was synthesized by stirring 0.537 g (2 mmol) NiBr_2_ in 50 ml iso­propanol at 330 K for ten hours. Green needle/lath-shaped crystals were obtained after slow evaporation of the solvent at room temperature.

### S2. Refinement

The carbon-bound H atoms were positioned with idealized geometries and were refined with C—H = 0.98 Å (methyl) and C—H = 1.00 Å (methine) and with *U*_eq_(H) = 1.2 *U*_eq_(C) using a riding model approximation. The H atom of the hy­droxy groups were initially found from a difference map and were refined with O—H distance restraints of 0.82 (2) Å and with *U*_eq_(H) = 1.2*U*_eq_(O).

### Crystal data

**Table:**

[NiBr_2_(C_3_H_8_O)_4_]	*F*(000) = 468
*M**_r_* = 458.91	*D*_x_ = 1.510 Mg m^−^^3^
Monoclinic, *P*2_1_/*c*	Mo *K*α radiation, λ = 0.71073 Å
Hall symbol: -P 2ybc	Cell parameters from 2876 reflections
*a* = 5.8341 (7) Å	θ = 2.3–24.6°
*b* = 10.4902 (15) Å	µ = 4.93 mm^−^^1^
*c* = 16.613 (2) Å	*T* = 199 K
β = 97.074 (4)°	Lath, green
*V* = 1009.0 (2) Å^3^	0.42 × 0.21 × 0.07 mm
*Z* = 2	

### Data collection

**Table:**

Bruker APEXII CCD diffractometer	1770 independent reflections
Radiation source: fine-focus sealed tube	1451 reflections with *I* > 2σ(*I*)
Graphite monochromator	*R*_int_ = 0.056
ω scans	θ_max_ = 25.1°, θ_min_ = 2.3°
Absorption correction: multi-scan (*SADABS*; Bruker, 2006)	*h* = −6→6
*T*_min_ = 0.305, *T*_max_ = 0.710	*k* = −12→12
9106 measured reflections	*l* = −19→19

### Refinement

**Table:**

Refinement on *F*^2^	Primary atom site location: structure-invariant direct methods
Least-squares matrix: full	Secondary atom site location: difference Fourier map
*R*[*F*^2^ > 2σ(*F*^2^)] = 0.029	Hydrogen site location: inferred from neighbouring sites
*wR*(*F*^2^) = 0.071	H atoms treated by a mixture of independent and constrained refinement
*S* = 1.01	*w* = 1/[σ^2^(*F*_o_^2^) + (0.0304*P*)^2^ + 0.450*P*] where *P* = (*F*_o_^2^ + 2*F*_c_^2^)/3
1770 reflections	(Δ/σ)_max_ < 0.001
94 parameters	Δρ_max_ = 0.60 e Å^−^^3^
2 restraints	Δρ_min_ = −0.34 e Å^−^^3^

### Special details

**Table:**

Geometry. All e.s.d.'s (except the e.s.d. in the dihedral angle between two l.s. planes) are estimated using the full covariance matrix. The cell e.s.d.'s are taken into account individually in the estimation of e.s.d.'s in distances, angles and torsion angles; correlations between e.s.d.'s in cell parameters are only used when they are defined by crystal symmetry. An approximate (isotropic) treatment of cell e.s.d.'s is used for estimating e.s.d.'s involving l.s. planes.
Refinement. Refinement of *F*^2^ against ALL reflections. The weighted *R*-factor *wR* and goodness of fit *S* are based on *F*^2^, conventional *R*-factors *R* are based on *F*, with *F* set to zero for negative *F*^2^. The threshold expression of *F*^2^ > σ(*F*^2^) is used only for calculating *R*-factors(gt) *etc*. and is not relevant to the choice of reflections for refinement. *R*-factors based on *F*^2^ are statistically about twice as large as those based on *F*, and *R*- factors based on ALL data will be even larger.

### Fractional atomic coordinates and isotropic or equivalent isotropic displacement parameters (Å^2^)

**Table:**

	*x*	*y*	*z*	*U*_iso_*/*U*_eq_	
Br1	0.71762 (5)	−0.08471 (4)	0.09301 (2)	0.04316 (14)	
Ni1	1.0000	0.0000	0.0000	0.02592 (16)	
O1	1.2185 (4)	0.0765 (3)	0.09534 (13)	0.0434 (6)	
H1	1.350 (4)	0.051 (3)	0.096 (2)	0.052*	
O2	0.8057 (4)	0.1654 (2)	−0.00752 (15)	0.0427 (6)	
H2	0.667 (3)	0.149 (4)	−0.020 (2)	0.051*	
C1	1.2416 (8)	0.0311 (5)	0.2387 (2)	0.0759 (14)	
H1A	1.1464	−0.0454	0.2276	0.091*	
H1B	1.2137	0.0674	0.2910	0.091*	
H1C	1.4051	0.0084	0.2406	0.091*	
C5	0.8588 (6)	0.2959 (3)	−0.0232 (2)	0.0486 (9)	
H5A	1.0299	0.3070	−0.0118	0.058*	
C2	1.1799 (6)	0.1270 (4)	0.17316 (19)	0.0447 (9)	
H2A	1.0116	0.1466	0.1713	0.054*	
C4	0.7475 (9)	0.3790 (4)	0.0349 (3)	0.0859 (16)	
H4A	0.8065	0.3557	0.0907	0.103*	
H4B	0.5797	0.3669	0.0263	0.103*	
H4C	0.7839	0.4686	0.0254	0.103*	
C6	0.7873 (8)	0.3295 (5)	−0.1100 (3)	0.0852 (16)	
H6A	0.8630	0.2718	−0.1449	0.102*	
H6B	0.8325	0.4176	−0.1197	0.102*	
H6C	0.6193	0.3210	−0.1223	0.102*	
C3	1.3116 (9)	0.2494 (4)	0.1882 (3)	0.0796 (14)	
H3A	1.2848	0.2843	0.2410	0.096*	
H3B	1.2591	0.3108	0.1454	0.096*	
H3C	1.4769	0.2330	0.1881	0.096*	

### Atomic displacement parameters (Å^2^)

**Table:**

	*U*^11^	*U*^22^	*U*^33^	*U*^12^	*U*^13^	*U*^23^
Br1	0.0251 (2)	0.0584 (3)	0.0462 (2)	−0.00264 (16)	0.00532 (15)	0.01303 (16)
Ni1	0.0212 (3)	0.0256 (3)	0.0302 (3)	−0.0008 (2)	0.0002 (2)	0.0006 (2)
O1	0.0280 (13)	0.0622 (17)	0.0388 (13)	0.0019 (12)	−0.0008 (11)	−0.0168 (11)
O2	0.0243 (12)	0.0292 (12)	0.0733 (16)	−0.0001 (11)	0.0006 (11)	0.0071 (12)
C1	0.080 (3)	0.093 (4)	0.053 (2)	−0.006 (3)	0.000 (2)	0.009 (2)
C5	0.034 (2)	0.030 (2)	0.080 (3)	−0.0017 (15)	0.0010 (19)	0.0072 (17)
C2	0.039 (2)	0.057 (2)	0.0371 (18)	−0.0003 (17)	0.0038 (15)	−0.0118 (16)
C4	0.077 (3)	0.048 (3)	0.135 (5)	0.008 (2)	0.023 (3)	−0.016 (3)
C6	0.078 (3)	0.075 (3)	0.098 (3)	−0.014 (3)	−0.006 (3)	0.043 (3)
C3	0.103 (4)	0.065 (3)	0.070 (3)	−0.020 (3)	0.009 (3)	−0.024 (2)

### Geometric parameters (Å, º)

**Table:**

Br1—Ni1	2.5532 (4)	C5—C6	1.493 (6)
Ni1—O2	2.068 (2)	C5—C4	1.506 (6)
Ni1—O2^i^	2.068 (2)	C5—H5A	1.0000
Ni1—O1	2.069 (2)	C2—C3	1.502 (5)
Ni1—O1^i^	2.069 (2)	C2—H2A	1.0000
Ni1—Br1^i^	2.5532 (4)	C4—H4A	0.9800
O1—C2	1.440 (4)	C4—H4B	0.9800
O1—H1	0.814 (18)	C4—H4C	0.9800
O2—C5	1.434 (4)	C6—H6A	0.9800
O2—H2	0.825 (18)	C6—H6B	0.9800
C1—C2	1.494 (5)	C6—H6C	0.9800
C1—H1A	0.9800	C3—H3A	0.9800
C1—H1B	0.9800	C3—H3B	0.9800
C1—H1C	0.9800	C3—H3C	0.9800
			
O2—Ni1—O2^i^	180.00 (12)	C6—C5—C4	113.0 (4)
O2—Ni1—O1	90.12 (9)	O2—C5—H5A	108.0
O2^i^—Ni1—O1	89.88 (9)	C6—C5—H5A	108.0
O2—Ni1—O1^i^	89.88 (9)	C4—C5—H5A	108.0
O2^i^—Ni1—O1^i^	90.12 (9)	O1—C2—C1	110.9 (3)
O1—Ni1—O1^i^	180.00 (15)	O1—C2—C3	109.3 (3)
O2—Ni1—Br1	86.49 (7)	C1—C2—C3	112.5 (3)
O2^i^—Ni1—Br1	93.51 (7)	O1—C2—H2A	108.0
O1—Ni1—Br1	93.12 (7)	C1—C2—H2A	108.0
O1^i^—Ni1—Br1	86.88 (7)	C3—C2—H2A	108.0
O2—Ni1—Br1^i^	93.51 (7)	C5—C4—H4A	109.5
O2^i^—Ni1—Br1^i^	86.49 (7)	C5—C4—H4B	109.5
O1—Ni1—Br1^i^	86.88 (7)	H4A—C4—H4B	109.5
O1^i^—Ni1—Br1^i^	93.12 (7)	C5—C4—H4C	109.5
Br1—Ni1—Br1^i^	180.000 (18)	H4A—C4—H4C	109.5
C2—O1—Ni1	132.8 (2)	H4B—C4—H4C	109.5
C2—O1—H1	111 (3)	C5—C6—H6A	109.5
Ni1—O1—H1	112 (3)	C5—C6—H6B	109.5
C5—O2—Ni1	133.0 (2)	H6A—C6—H6B	109.5
C5—O2—H2	112 (3)	C5—C6—H6C	109.5
Ni1—O2—H2	111 (3)	H6A—C6—H6C	109.5
C2—C1—H1A	109.5	H6B—C6—H6C	109.5
C2—C1—H1B	109.5	C2—C3—H3A	109.5
H1A—C1—H1B	109.5	C2—C3—H3B	109.5
C2—C1—H1C	109.5	H3A—C3—H3B	109.5
H1A—C1—H1C	109.5	C2—C3—H3C	109.5
H1B—C1—H1C	109.5	H3A—C3—H3C	109.5
O2—C5—C6	111.1 (3)	H3B—C3—H3C	109.5
O2—C5—C4	108.4 (3)		

Symmetry code: (i) −*x*+2, −*y*, −*z*.

### Hydrogen-bond geometry (Å, º)

**Table:**

*D*—H···*A*	*D*—H	H···*A*	*D*···*A*	*D*—H···*A*
O1—H1···Br1^ii^	0.81 (2)	2.58 (2)	3.372 (2)	166 (4)
O2—H2···Br1^iii^	0.83 (2)	2.51 (2)	3.315 (2)	165 (3)

Symmetry codes: (ii) *x*+1, *y*, *z*; (iii) −*x*+1, −*y*, −*z*.
