# Supplementary figures and images for: Crystal structure of di­bromido­tetra­kis(propan-2-ol-κO)nickel(II)
Source: Acta Crystallogr E Crystallogr Commun. 2015 Dec 19;71(Pt 12):m263–4. doi: 10.1107/S2056989015023555 (PMC4719865; doi:10.1107/S2056989015023555)

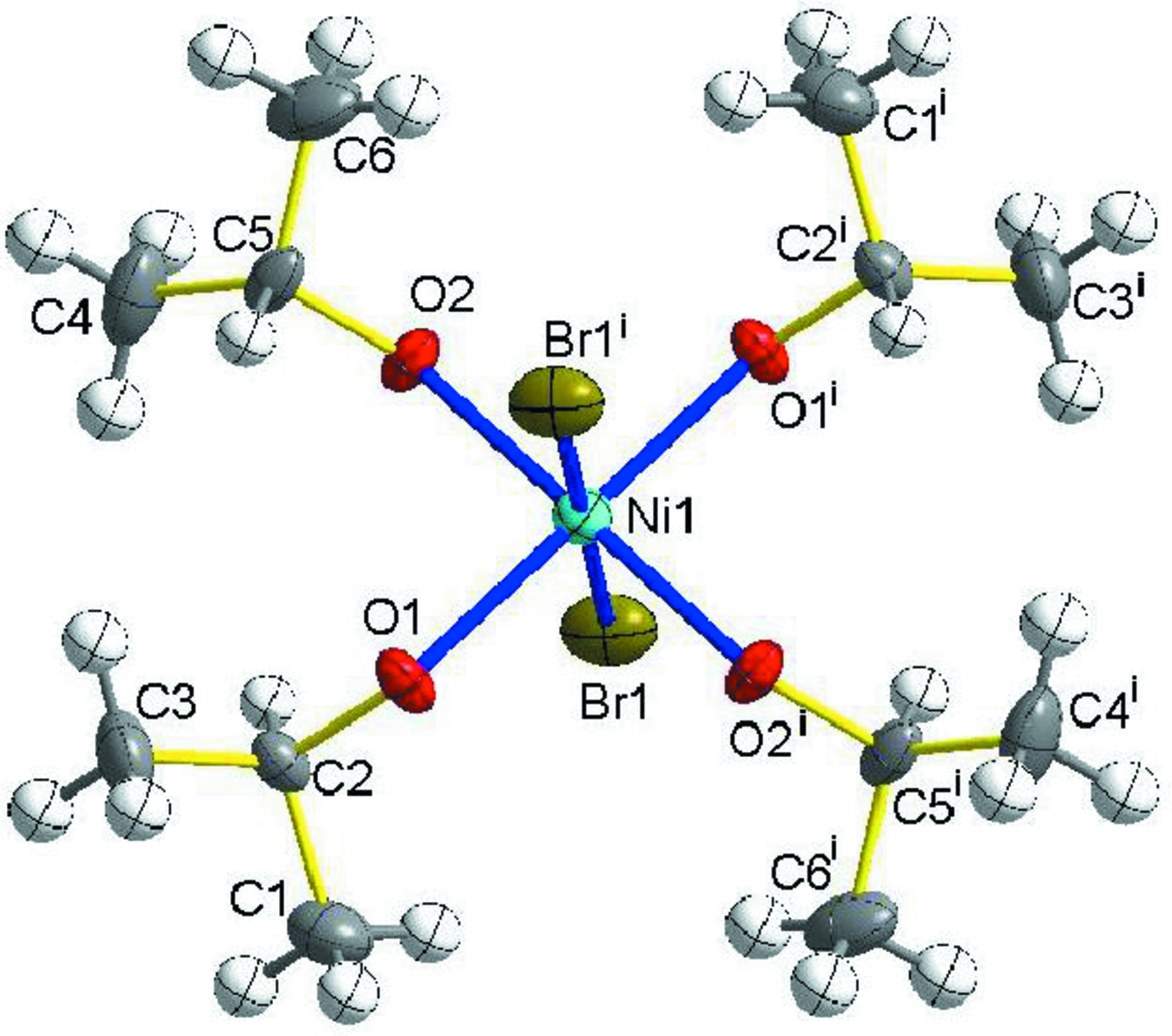

Supplement: Supplementary file 3 [file e-71-0m263-fig1.tif]

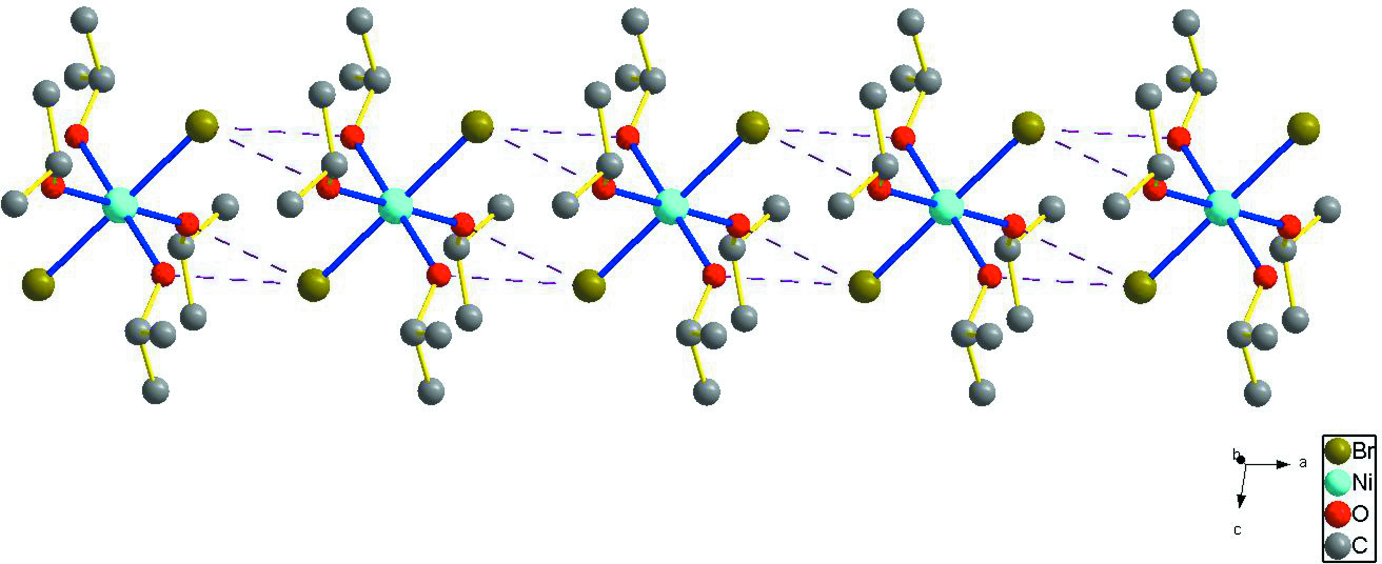

Supplement: Supplementary file 4 [file e-71-0m263-fig2.tif]
